# Strengths and Challenges of the Speech and Language Therapy (SLT) Degree Apprenticeship Route: Initial Stakeholder Perspectives

**DOI:** 10.1111/1460-6984.70071

**Published:** 2025-07-07

**Authors:** Hazel Richards, Victoria Lundie

**Affiliations:** ^1^ Health, Education and Life Sciences Birmingham City University Birmingham UK

**Keywords:** collaboration, degree apprenticeship, mentor, speech and language therapy, work‐based‐learning

## Abstract

**Background:**

This article shares details of a pilot research project that explored stakeholder perceptions and experiences of the academic and work‐based elements involved in one university's innovative speech and language therapy (SLT) degree apprenticeship.

**Aims:**

To share findings about SLT apprentice learner and mentor expectations and experiences since this knowledge, including of barriers and facilitators, will enable the university concerned, and others already providing or developing their SLT degree apprenticeship, to enhance their offer and so apprentice learning experience and outcomes.

**Methods and Procedures:**

Following ethical approval, a mixed‐methods research design was applied. In phase one, 18 apprentice and mentor participants involved in the programme completed online questionnaires. Themes identified from inductive thematic analysis of the questionnaire data were probed further in nine online semi‐structured interviews, with transcripts also being analysed using inductive thematic analysis.

**Outcomes and Results:**

Findings offer new knowledge about the apprenticeship route into the profession and provide valuable insight for HEIs and employers considering or developing an SLT or wider allied health profession (AHP) apprenticeship offer. This includes detail about the strengths and challenges related to four key themes: internal factors; time and support; structure and organisation; and apprenticeship processes.

**Conclusions and Implications:**

The apprenticeship provides an economically sustainable and practical progression opportunity for learners, including SLTAs, and may provide SLT training that is better embedded in clinical practice than traditional routes. Collaboration between the key stakeholders (apprentice learner, workplace mentor, and HEI) is central to this, though challenges in terms of time and resources exist. Developing and sustaining SLT apprenticeships therefore has implications for future workforce development and the profession.

**WHAT THIS PAPER ADDS:**

*What is already known on this subject*

- Degree apprenticeships, as a route into the speech and language therapy profession, are in their infancy; thus, research and information about them are emergent.

*What this study adds to the existing knowledge*

- The university where the study took place had been involved in the RCSLT apprenticeship trailblazer group since its inception in 2017 and offered the first full‐time SLT Degree Apprenticeship (DA). Findings about what is different about SLT DA education and learners as compared to traditional routes are presented. The study provides new information about who chose to study SLT via the DA and why, and what components should be considered, including how, when and where DAs might be delivered.

*What are the clinical implications of this study?*

- Whilst the DA SLT appears to have distinct advantages over more traditional routes in terms of continual integration of theoretical and work‐based learning and experience, challenges around workload exist. This study was conducted at an early stage of the DA delivery, so knowledge about threats and whether the route will fulfil workforce development hopes remains to be seen.

## 1 Introduction

### 1.1 Why a Degree Apprenticeship (DA)?

Allied health professions (AHP) workforce planning in England, including the Speech and Language Therapy profession, identifies the need for routes into professions that are sustainable, more economically viable for learners, and which facilitate more representative participation (RCSLT 2022). Apprenticeships are seen as a route that offers quality and social mobility, but which require pedagogical innovation and careful navigation (Crawford‐Lee and Moorwood 2019). DAs are a new route into the profession, so research specific to SLT apprenticeships is limited to date. However, literature about the apprentice route for nurses introduced in 2017 (Derbyshire et al. 2024), and other, more recently introduced AHP apprenticeships such as radiography (Sevens et al. 2022) and occupational therapy (Liddell et al. 2023) is available. Such literature identifies that benefits and challenges (Mulkeen et al. 2019; Quew‐Jones 2023) are both inherent to this route.

### 1.2 Benefits and Challenges

Hiring apprentices is an effective skill acquisition strategy, and employers are embracing the route as a strategic tool to build a loyal, dedicated and motivated workforce (Antcliff et al. 2016; Smith et al. 2021), though this does require careful management by the employer (Rowe et al. 2017; Roberts et al. 2019). DAs offer learners a potentially transformative opportunity (Derbyshire et al. 2024) and enhanced employability as well as a chance to earn whilst learning (Smith et al. 2021).

### 1.3 Who Are the Main Stakeholders?

In addition to apprentice employers and learners, universities and professional bodies are also key stakeholders in AHP apprenticeships. Most NHS organisations meet the apprenticeship levy threshold of a staffing budget of more than £3 million, so can use this to fund apprenticeship education, including DAs (Gov.uk 2016). The government also contributes 90% of the funding for small‐medium enterprises (SMEs) (Gov.uk 2018). This has created opportunities for Higher Education Institutes (HEIs) and fits with university inclusivity strategies, with higher apprenticeships continuing to grow (106 360 in 2021/2022, 112 930 in 2022/2023, 130 830 in 2023/2024 and 132 560 in 2024/2025, Gov.uk 2025). However, Crawford‐Lee and Moorwood (2019) suggest these opportunities need to be set against the competitive challenges of apprenticeship delivery compared to traditional HEI provision. Certainly, this opportunity requires strong institution–employer partnerships, integration between work‐based and academic learning, multiple workplace learning opportunities, co‐ordination of assessment, and quality assurance (Lester 2020) as well as stakeholder collaboration and work‐integrated learning (Quew‐Jones 2023). So, whilst aspects of traditional undergraduate and apprentice routes are similar, for example, academic content, assessment, and the need to align to occupational standards (Council of Deans of Health 2023), there are also key differences between the two routes.

### 1.4 What Is Different From a Traditional Undergraduate to an Apprenticeship Route?

AHP apprenticeship providers must plot workforce progression against their professional body training and curriculum requirements (RCSLT 2017, 2021), against the relevant profession's apprenticeship learning knowledge, skills and behaviours (KSBs) (Institute for Apprenticeships 2023) and address the Health and Care Professions Council (HCPC) Standards of Proficiency (2023), with the concept of KSB's being new to most HEI educators.

Apprentice learners (ALs) often hold valuable experience, and because they are employed in work related to their topic of study, their work‐based‐learning (WBL) contributes a significant amount to their development. Konstantinou and Miller (2020) argue a reflective approach in assessments together with an explicit focus on skill development can enhance degree apprentices learning. Garnett (2020) suggests this requires innovative curriculum design, which in the institution concerned, has involved mapping WBL onto six level descriptors for experiential learning (exposure, participation, identification, internalisation, dissemination, and equality, diversity and inclusion, Steinaker and Bell 1975).

ALs may be proficient in their jobs, and must destabilise their own identity as they become novices in their field of study and gradually increase their knowledge, skills and capabilities (Derbyshire et al. 2024; Martin et al. 2020), a process that their university tutors and work‐place mentors also undergo (Martin et al. 2020; Roberts et al. 2019). In addition to the tasks of learning new systems and academic skills (Saville et al. 2020) that face all undergraduates, ALs must also learn to balance the demands of work and study (Bravenboer and Lester 2016) as well as placement so they develop excellent time‐management skills (Baker 2019). A further challenge relates to communities of learning (Wenger 2015), which in the ALs case involves different communities with differing dynamics (workplace professionals, including mentors, peers and university) (Nottingham and Yan 2023). The means by which the degree apprentice learns to become a competent professional in practice is therefore more complex than for traditional undergraduates, with workplace mentors holding a key role. This includes helping the ALs understanding of theory‐practice links, setting workplace expectations of professionalism and facilitating learning within and outside the workplace (Roberts et al. 2019). Another difference is the tri‐partite system, an integral part of the apprenticeship process. Taylor‐Smith et al. (2023) identified that whilst regular tripartite meetings are a requirement, their meaningfulness and impact are enhanced by positive relationships. The university plays a key role in facilitating these, and apprentices play a pivotal role in sharing knowledge and skills bi‐directionally between academia and the workplace. The literature therefore identifies the importance of stakeholders working collaboratively to develop apprenticeship programmes.

### 1.5 Why Is It Important to Find Out/Work Collaboratively to Develop This Programme?

In their case study of two employers in the field of digital technology, Antcliff et al. (2016, 381) identify that DAs require ‘*substantial investment in an employee—… but it's worth it*.’ This is due to opportunities to contribute to the learning of their employee and because of the contributions the apprentice makes to the workplace, though strong relationships, trust and ongoing dialogue between stakeholders are key to fulfilling the needs of employers. Bravenboer (2016) comments on an unhelpful distinction between academic learning and on‐the‐job training but also recognises the expertise universities can bring to designing and assessing higher‐level learning. Implementation of a DA programme for the first time is a complex and time consuming process requiring commitment from all stakeholders. Whilst some of this needs to be done before an apprenticeship programme is implemented, other tasks are ongoing. Successful completion is linked to well‐structured and comprehensive support whilst on the programme (Baker 2019), with employers playing an important role in positive transitions into graduate employment (Jones et al. 2023). Voeller (2023) suggests that this involves four interconnected dimensions: a well‐designed curriculum; alignment of university and workplace learning; reinforcement through real‐world application; and support by ongoing mentoring, which involves both formal and informal mentoring. Importantly, Voeller (2024, 43) describes mentoring training as ‘*an essential service*,’ with Minton and Lowe (2019) suggesting the facilitation of on‐the‐job learning requires purposeful outlining, benefits from being delivered in a structured, consistent, and sustained way, and involves acknowledgement of tensions between short‐term productivity and sustainable development.

## 2 Potential Challenges to Consider

Achieving all of this is not without challenge. Whilst collaboration between HEIs and employers is welcome, Welbourn et al. (2019) comment on the reality of university responsiveness to new curriculum models for workforce development. Quew‐Jones (2023) identifies how programmes offered by multiple disciplines and providers differ widely, but proposes governance is possible through a knowledge‐based checklist aimed at strategic, academic department, and teaching levels. Others identify that the different processes and structures involved in apprenticeships make new demands on academic staff, and can even lead to questions about whether this is ‘proper academic’ work (Martin et al. 2020). This is interesting and can be juxtaposed with the 21st‐century context where apprenticeships are no longer just the choice for individuals who cannot go to university. Rather, they are a highly aspirational and pragmatic choice, with DAs providing parity of status and the same opportunity as traditional degree routes (Mulkeen et al. 2019).

This pilot study explored stakeholder perceptions and expectations of the SLT DA after its first year. It investigated the programme at this early stage of delivery with an objective of using the knowledge gained, including of barriers and facilitators, to enhance the apprenticeship offering and learning experience at the HEI where the study was conducted. As this is a trailblazer course, this knowledge will identify specific needs of SLT degree apprentices, including strategies to attract and support this new population, and will also contribute to the development of apprenticeships in other AHP professions and HEIs.

## 3 Methods

### 3.1 Aims

The university where the research took place had been involved in the RCSLT apprenticeship trailblazer group since its inception in 2017, offering the first full‐time SLT DA which commenced in January 2023. The project aimed to find out about SLT apprenticeship learner and mentor expectations, experiences and aspirations with an objective of using the knowledge gained, including of barriers and facilitators, to enhance the apprenticeship offering and learning experience at the HEI where the study was conducted. Three research questions were investigated:

1. What are stakeholders’ (apprentices and work‐based mentors) expectations of the SLT DA programme at this early stage of delivery?
2. What are stakeholders’ experiences of this HEI's SLT DA programme at this early stage of delivery?
3. What are stakeholders’ aspirations for SLT DA completers?

### 3.2 Methodology

This research sought to access individual experience and perspectives. Although some numerical data were collected, a predominantly interpretivist paradigm was adopted (Blaikie and Priest 2019). Interpretivists use a mix of both qualitative and quantitative methods to help them understand the subject being studied. This is because different methods reach various aspects of the subject, though the emphasis tends to be on qualitative data. This research used online questionnaires to capture both numerical (about participant demographics) data, for example, level of training, and descriptive data, for example, experience of the work/study balance. The themes identified by analysis of this first data set informed the semi‐structured interview questions—used in the second phase to probe the questionnaire findings in more depth.

### 3.3 Ethical Approval

A detailed application for ethics approval was made to the faculty Ethics Committee. This included ensuring total anonymity of apprentice participants to remove any possibility of bias or the presence of power differentials. As participants were required to complete consent forms, these were administered and stored in a secure HEI OneDrive folder set up by a research assistant (RA) (*n* = 1) not involved in the apprenticeship programme. Links to the online questionnaires (either mentor or learner versions) were sent by this first RA, who served as a research administrator, with the online questionnaire platform assigning numerical participant codes. All other RAs (2 for questionnaire analysis, and 3 to conduct, transcribe and analyse the interviews, *n* = 5) were Level 6 traditional route SLT students, who had no prior knowledge or contact with the apprentice or mentor participants and who are versed in professional codes of conduct, including maintaining confidentiality (HCPC 2024). This ensured anonymity (no staff member knows the identity of any participant), minimised participant vulnerability and prevented any conflict of interest.

### 3.4 Sampling Procedures

Learners who are currently SLT degree apprentices and their work‐based mentors, were recruited via convenience sampling. This is a distinct group which consists of 19 of each of the two participant groups described above. Eighteen questionnaire responses were received from 12 ALs and six mentors. Tables 1 and 2 show demographic information for these:

**TABLE 1:** Demographic information of apprentice learner (AL) questionnaire participants.

Area of employment	NHS *n* = 10	Independent *n* = 2	Charity *n* = 0			
Existing employee of the organisation supporting learner through apprenticeship?	Yes *n* = 11	No *n* = 1				
Hold an existing degree?	Yes *n* = 5	No *n* = 7				
Role prior to apprenticeship	SLT assistant *n* = 8	Teaching assistant *n* = 1	Early years health Practitioner n = 1	Rehabilitation assistant *n* = 1	Primary school tutor *n* = 1	
Clinical area worked in	Early years *n* = 3	Education, paediatric *n* = 3	ASD pathway *n* = 1	Adult acquired, community *n* = 2	Adulte acquired, acute *n* = 1	Adult mental health, acute *n* = 2

**TABLE 2:** Demographic information of mentor questionnaire participants.

Area of employment	NHS *n* = 5	Independent *n* = 0	Charity *n* = 1	
Ring‐fenced time for mentor role?	Yes *n* = 2	No *n* = 4		
Role with apprentice	Mentor only *n* = 2	Line manager *n* = 0	Mentor and Clinical Supervisor *n* = 3	Mentor and placement coordinator *n* = 1
Recruitment to mentor role	Previous role supervising SLTA *n* = 3	Asked to *n* = 2	Volunteered to *n* = 1	

Five ALs and four mentors, recruited from the 18 questionnaire participants detailed above, volunteered to take part in the semi‐structured interviews (*n* = 9).

### 3.5 Questionnaires

The first phase of data collection used online questionnaires, chosen as being accessible to each group. Although response rates to questionnaires can be lower, they allow anonymity to be preserved, can be easily distributed, and allow participants to complete them at a time most convenient to them (Braun et al. 2021). A combination of closed, ranked and open questions was included in both the AL and mentor questionnaires to find out demographic information about participants and about their experience prior to becoming involved with the apprenticeship, their current experience of being involved with the apprenticeship, and detail around available support, the course itself, placements; and finally, their ideas for the future. This was administered in October‐November 2023, using Jisc, which is GDPR (General Data Protection Regulation) compliant.

### 3.6 Semi‐Structured Interviews

The second phase of data collection, involving individual semi‐structured interviews, was conducted in January–February 2024. These probed and extended understanding of the themes obtained from the questionnaire analysis. Semi‐structured interviews were chosen, as they allow for deeper and synchronous investigation of an individual's experience and perceptions of a phenomenon, whilst being structured enough to enable comparisons across participants. The semi‐structured interviews were conducted online to allow for a greater reach of participants (who were widely spread geographically) and to facilitate transcription through Microsoft Teams. Duration ranged from 20 to 45 min. Interview recordings were downloaded from Microsoft Teams, onto a university encrypted laptop, then erased from Microsoft Teams. Interview transcriptions were downloaded, edited and anonymised, then returned to interviewee participants for member checking. ALs were asked 10 questions, for example about managing work, study and university days; the WBL portfolio; academic support; identity development; work‐life balance; and peer group support. Mentors were asked eight questions, for example about what supports and equips them as mentors; the WBL portfolio; tri‐partite meetings; the organisation of placements; monitoring apprentice progress; and time management.

### 3.7 Approach to Data Analysis

**FIGURE 1:**
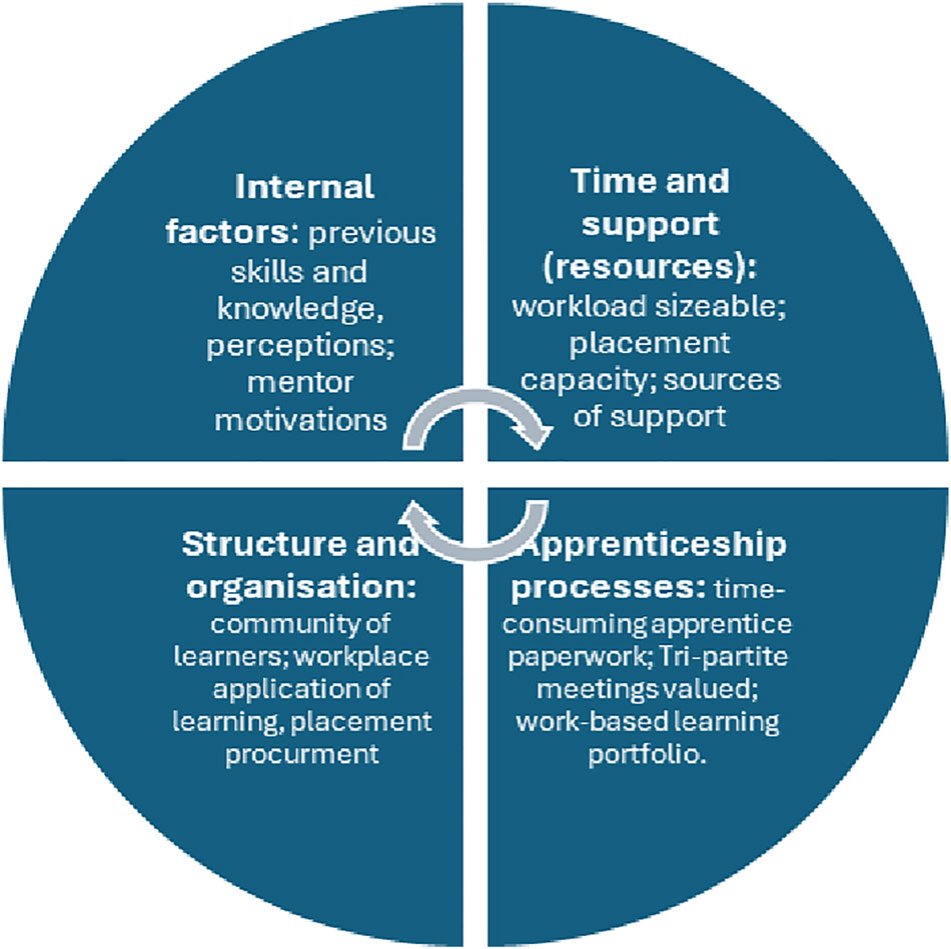
Themes and subthemes identified from apprentice learner (AL) and mentor data.

Thematic analysis was used as the paradigm for analysis (Braun and Clarke 2021). This consists of six distinct stages: familiarising yourself with the data; data extraction; generating initial codes; collating codes into potential themes; reviewing themes to check if they work in relation to the coded extracts and entire data set; then refining these and generating a thematic map. An inductive (data‐driven) approach was chosen to reduce researcher bias by enabling the RAs, who had no previous contact with the participants, and less knowledge of the course, to identify key themes from within the data. However, thematic analysis recognises the role researchers have in developing themes, including how their knowledge and experience shape these (Braun and Clarke 2022). The research investigators therefore became involved at the last stage of analysis only, discussing the codes and themes identified by the RAs to understand their thought process, and using their data reduction grids (consisting of codes, themes, and illustrative quotes) to inform the conceptual model (see Figure 1) and report results.

The Jisc online platform used to administer the questionnaires collated quantitative question responses and generated descriptive statistics. The phases identified for thematic analysis (Braun and Clarke 2021) were adhered to for both qualitative questionnaire response analysis (phase one) and interview transcript analysis (phase two). RAs created data extraction tables comprising codes and all relevant quotes, discussing emerging themes and agreeing on/modifying key themes. These tables were then used by the research investigators to conceptualise and evidence the overarching themes.

Braun and Clarke (2022) highlight the need for a reflective, rigorous, systematic and thorough approach, which can be achieved by addressing the four key strategies highlighted by McAllister and Lyons (2022). Credibility (internal validity) was achieved at a participant and stakeholder debriefing event, when the findings were shared and discussed. Confirmability (objectivity) was achieved by using RAs not involved with the DA to code the data. The data reduction table (Malterud 2012), where RAs included a participant quote for every item coded, ensured every code could be illustrated and contextualised. The inclusion of participant statements that contrasted with the majority, for example, that the apprenticeship contained ‘less theory’ than traditional academic study, ensured themes were consistent with views expressed by all participants (Robson and McCarten 2016). Dependability, or inter‐rater reliability, was achieved through the discussion's RAs held to check and agree on meanings and to jointly identify examples of these within the transcripts. The RAs and research investigators then met to provide peer scrutiny, reading transcripts and checking codes and related participant quotes for external credibility.

Collaborative research investigator discussions then allowed for reflexive, collective decision‐making regarding the convergence of the AL and mentor themes and reporting of findings, delivered through poster and spoken presentations at internal events, and externally with the RCSLT apprenticeship trailblazer group. These aimed to increase transferability, with thick description (McAllister and Lyons 2022) through the inclusion of participant quotes, enabling interested parties to contextualise the findings in relation to their own contexts.

## 4 Findings

Four overarching themes were identified from AL and mentor data:

### 4.1 Internal Factors Strengths

ALs identified how previous jobs and employment provided gateways to the apprenticeship in the form of experience and by clarifying SLT as their chosen career. Eight out of 12 questionnaire AL participants had been SLT assistants and viewed the DA as a way to progress their careers, and one AL had chosen this route as an alternative to studying SLT as an MSc. Four out of five interview AL participants perceived the apprenticeship as more practical than a degree route, valuing the blend of academic and practical work. The monetary aspect was important for four out of the five AL interview participants. For example, ‘*family circumstances meant it just wasn't possible for me to do a BSc and give up working*’ (AL5) and ‘*because I could not afford to not work to do the masters, so it was my route*’ (AL3). Another had specifically waited for the apprenticeship to become a reality, since this route meant they were ‘*able to run my house and get qualified at the same time*’ (AL4). Only one AL commented on how the apprenticeship had or would change their identity, stating: ‘*it's added to my identity, you know, like I'm doing something different which will ultimately change or, you know, advance my professional life*’ (AL5). Most ALs felt they would probably stay with their current employer after qualifying, though they felt it was too soon to say if they might move jobs or clinical areas in the future. Overall, the apprenticeship was seen as an exciting opportunity, and ALs were proud of being the first cohort and motivated despite university days requiring some of them to get ‘*up at half four every Monday morning*’ (AL3), or ‘*travel three or four hours to get to university*’ (AL5).

All four mentors interviewed felt the apprenticeship is important, citing reasons including ‘*fantastic people can now train to be a SLT through an apprenticeship because it is such a vocational career*’ (M1); ‘*it's gonna help workload in the future where retaining workforce, valuable members of staff that would perhaps otherwise go out elsewhere because they're not being pushed and not being developed*’ (M2); and ‘*I think it's quite innovative and probably the way the profession's going. So, I kind of wanted to get on there nice and early and learn about it as much as I could. I did a careers day a couple of months ago, and people, I'd say 90% of the people coming up to me were asking ‘can I do this as an apprentice rather than wanting to go through the university route?’ I think particularly now you have to pay for the degree*’ *(M4)*. All enjoyed the role, identifying mentoring as being ‘*quite different to being a placement practise educator*’ (M1) due to the length of the training journey. They valued their relationship with their apprentice, and felt apprentices needed a different approach, for example more like coaching where ‘*you are teasing ideas out of them*’ (M4). They recognised their own skills had developed due to the mentor role, with one (M4) recognising how skills learnt from mentoring would help in their ultimate aim of becoming a clinical team leader. Finally, mentors expressed a sense of pride in their apprentice, were very positive about the apprentices’ skills, felt responsibility for their apprentice to succeed, and aspired to be an inspiration, for example: ‘*I want to be like the role model to be the speech therapist she one day wants to kind of be herself. That's the ultimate aim*’ (M2) and ‘*I would just love to inspire [Apprentice] to be an amazing therapist and to kind of stick at it because it's tough training*’ (M1).

### 4.2 Internal Factors Challenges

In contrast to these strengths, one AL wondered ‘*whether actually we're getting enough information. So, like with the undergrad. or the masters, it's a lot more teaching time and a lot more work. And then I'm thinking, are we getting enough of the theory …I still wonder*’ (AL3). Another felt the term ‘apprenticeship’ can have negative connotations: ‘*for me I think apprentice or apprenticeships is something that you know, kind of school leavers do. And so, there's that connotation to that you're very young, that you're very much learning and it's interesting actually because like obviously I've been in the job for quite a while. The view of other people of you is different*’ (AL5).

### 4.3 Time and Support (Resources) Strengths

Time and support strengths included a helpful and cohesive university peer group which shared knowledge, advice, and motivation. The social experience and feeling of community that face‐to‐face university days have created were valued for alleviating the sense of isolation sometimes experienced. For example: ‘*that's been invaluable at times because I think it can feel quite isolating … you're at work and you're the only one doing it. You're the first people to be doing it and it's all so busy and so fast paced it can feel quite isolating if you're not connecting with those people that are kind of in the same situation*’ (AL5). Previous study (when present) helped ALs academic skills, as did support available in the HEI, though this was not always accessible given their working hours and responsibilities. Feedback from professionals in their workplace was recognised as supporting their development, and mentors were valued for providing a balance of professionalism, care and autonomy; their experience and interprofessional connections; being accessible; and being flexible to AL needs, though ALs recognised mentors are busy and must balance mentoring with other commitments.

Prior planning of ALs workload helped mentors manage their time, and receiving as much notice as possible about training and meetings is essential. Support from their own SLT and multi‐disciplinary‐teams was valued, including from managers and clinical supervision. University support and resources that were valued included Mahara (a training and resource site), drop‐ins, and guidance from lecturers who are accessible and approachable with M1 saying: ‘*I've felt in really safe hands, really, with having an apprentice at [names HEI]. I've kind of felt like, although I've said there's bits of information that maybe I've not been aware of or missed, I feel like things have been there. And if I've needed to ask something, it's been responded to really quickly*.’ Experiences of supervising traditional route students were drawn on though there are differences, for example, ‘*you only get them* [traditional route students] *for a short period of time and it's quite hard to fit in all the opportunities you want with them, whereas with the apprenticeship it's been quite nice to kind of be able to show her the real side of the job, with all the kind of meetings and everything like that*’ M2. Finally, training specific to the apprenticeship was valued, though pertinent information from the university did not always go directly to mentors or arrive with a good amount of notice. Mentors would also value a quick guide on general/essential expectations for their role, feeling expectations when hosting an apprentice were not clear, particularly with regard to changing university days over the duration of the apprenticeship, which is important to know for planning purposes, academic deadlines, and paperwork and processes.

### 4.4 Time and Support Challenges

Time and support challenges for ALs related to the intensity of the apprenticeship, with the workload identified as being considerable. ALs felt the university had unrealistic expectations of them regarding available time, for example, when reading takes longer than indicated or because of work pressures. They were concerned a lack of time could affect their grades, though they recognised self‐discipline supports role switching and that they had quickly learnt to create a structure to manage their time and submissions. For example: ‘*I’m not gonna lie ….juggling that I think for me the biggest thing is switching mindset between being in a sort of Uni mindset and then coming back. And that's Monday and Tuesday. And then coming in on Wednesday into the work I'm trying to get back into a work sort of frame of mind, always feel that takes near like half a day to a full day to then shift back into that’’* (AL4) and: ‘*it takes a lot of planning, a lot of like discipline … of being quite clear about what time is for what and when. I'm trying to manage that alongside just maintaining your own mental wellbeing and not getting ill stuff like that. I'm not sure if I've completely managed to get that down to a fine art—it's definitely a work in progress*’ (AL5).

Only two mentors had ring‐fenced time, and only two had no additional roles (see Table 2). No mentor sampled in this study (*n* = 6) had backfill, with all needing to prioritise their normal workload due to the impact it could have if they did not. The time they mentored their AL varied from 1–3 h per week, and being at the same site as their AL enabled them to provide ad‐hoc support when needed. However, lecturers are not always able to resolve/dispel mentor worries, and drop‐in sessions were not always at convenient times.

### 4.5 Structure and Organisation, Including Placement

ALs recognised benefits to working and studying simultaneously. They felt the structure of university, study and work‐based days was balanced, and the opportunity to earn whilst learning was valued, though managing all three components of the course (work, study and placement) was not easy. At this stage in the programme, ALs have only attended 6 separate placement observation days, which may explain why placements did not appear prominently in their responses.

Mentors identified challenges around placement, particularly around hosting traditional route students as well as apprentices, so were ‘*concerned about their capacity and their time to be able to support additional students*’ (M2). Mentors were not directly involved in procuring AL placements and would like more information/training, including around expectations for their role during placements. For example, M2 said ‘*I don't have a full picture, and I don't know whether it's because I don't need any of the picture or whether I need all of it, but just getting bits of it sometimes is a little bit stressful*.’ Mentors also had worries about how future placements could affect the workplace; for example, M1 stated, ‘*it needs just a little bit more careful planning really*.’

### 4.6 Apprenticeship Processes

This theme is concerned with apprenticeship‐specific processes. ALs felt the off‐the‐job log (detail of how university and study day hours have been spent for tracking purposes) was too time consuming and study days did not contain enough time for this. Some ALs found tri‐partite meetings to be supportive, whereas others described them as an unnecessary formality that feels like ‘*a tick box exercise which can be quite frustrating*’ (AL5), though there was recognition this would change as the apprenticeship progressed. Mentors, in contrast, appreciated tri‐partite meetings, and were prioritising them because they recognised them to be a source of information and support, and that they ‘*are a really good marker of progress*’ (M2). Mentors valued the way worries can be addressed in them, though also identified the need for ‘*a glossary of terms because …there are lots of kind of acronyms and lots of kind of quite jargony terms thrown around that [Apprentice] understands because she's on the course but I'm not always quite sure what they actually mean*’ (M4).

Comments on the WBL portfolio (a continuous record of workplace learning and achievement), which had only recently been introduced at the time of data collection (January–February 2024), were prevalent. Some ALs felt the WBL portfolio lacked clarity and accessibility, due to the document size and wording used, and that it was too time consuming to complete. However, there was recognition it was already becoming more useful, as it had practical relevance to their work and could be used to structure discussions and identify clinical experience gaps and opportunities. Mentors felt the WBL portfolio was a valuable resource, especially when it was a collaborative effort, for example: ‘*we've set up our own little team on Microsoft Teams and we've got it saved as a file. So, we both just annotate it as and when and then we kind of revisit it together. So that works really well for us*’ (M1). Mentors identified ambiguous wording and of being initially ‘*confused in terms of how you show the progression on it*’ (M2) but had used university drop‐in Q and A sessions and tri‐partite meetings to clarify this. Mentors also felt progress along the 5 columns (exposure, participation, identification, internalisation, dissemination, Steinaker and Bell 1975) was not linear for this first group of ALs, stating, for example, ‘*she's almost like too experienced for some of the columns, they should already be ticked off*’ (M3).

## 5 Discussion

This pilot study collected detail about the expectations, experiences and aspirations of a small group of ALs and mentors. Findings provide information about the strengths and challenges of one HEIs SLT DA offer, including detail about who chose to study SLT via the apprenticeship and why, and what components should be considered, including how, when and where DAs might be delivered.

### 5.1 Summary of the Findings

In summary, these participants saw the SLT apprenticeship as being a more practical route into the profession for them than a traditional degree, and increased queries about the apprenticeship as a route into SLT at career events were reported. This is because of the work‐study balance, valued for the opportunity it gives for ongoing application of learning. It is also because the apprenticeship offers an affordable way to study. This is despite negative connotations attached to the term ‘apprenticeship’ and perceptions that the course might contain less theory/content than traditional courses. The intensity and workload involved in the apprenticeship were recognised, with time management, skills learnt in previous study, and social and academic support obtained from peers and university identified as being important. Capacity issues, including impact on workload and traditional placement responsibilities, were also identified by mentors. Specific apprenticeship processes are time consuming, but tri‐partite meetings were appreciated as sources of support and information, and the WBL portfolio guides and informs ALs WBL opportunities. Information about stakeholder expectations of the apprenticeship was limited, perhaps because this is the first cohort. Although aspirations were not present, as it was ‘*too soon to say*,’ mentors aspired to be good role models, and valued the role for how it was progressing their own skills, CPD and career progression.

### 5.2 Integration With Previous Research

Findings from this study align with literature about other DAs. Key issues to consider in relation to effectiveness and sustainability (Lester 2020; Minton and Lowe 2018) were identified, as was detail pertaining to the dimensions Voeller (2023, 232) suggests must be considered when designing and delivering DA programmes, especially aligned university and workplace learning; learning reinforced through real‐world application; and relational aspects, where the apprentice is supported by ongoing mentoring.

### 5.3 Who and Why—Internal Factors

Literature identifies DAs offer a chance to earn whilst learning (Smith et al. 2021), a finding borne out in this study. Although five of the 12 AL participants already held degrees, ALs and mentors identified the apprenticeship as a powerful progression route for, for example, SLTAs who would not have had the same opportunities to progress otherwise. Certainly, this SLT apprenticeship provides opportunities to accrue, apply and share professional learning in a more dynamic way than traditional routes. (Nottingham and Yan 2023), which has advantages for adult‐learning, workforce development and clinical proficiency. However, it was too early in the programme to identify how loyal this group of ALs will be to their employers on completion (Antcliff et al. 2016; Smith et al. 2021). Although the findings highlight the vital role mentors play, including advantages apprenticeship mentoring has for mentor identity and development, challenges, are present (Roberts et al. 2019; Voeller 2024). For example, although we know strong relationships, trust and ongoing dialogue between stakeholders (Antcliff et al. 2016) are key, we don't yet know enough about the impact stakeholder salience and commitment have (Quew‐Jones 2023) on outcomes and sustainability.

### 5.4 What—Time and Support; Apprenticeship Procedures

Time was in short supply and needed careful management (Rowe et al. 2017; Roberts et al. 2019), with other AHP DA literature identifying the need to meet workplace/load requirements (Sevens et al. 2022) alongside the ‘substantial’ time investments involved in supporting apprentices (Antcliff et al. 2016, 381). Sources of support were important, though they differed for ALs and mentors. Baker (2019) states successful AL completion relies on well‐structured and comprehensive support whilst on the programme, and Voeller (2024) describes mentor training as being essential. The SLT DA requirements and progress have been carefully mapped against RCSLT (2017, 2021), HCPC (2018, 2023) and Institute of Apprenticeship (2023) standards, but most apprenticeship concepts and processes were new to HEI staff, ALs and mentors. This meant all three stakeholder groups were novices in this initial stage of delivery (Martin et al. 2020; Roberts et al. 2019). Tri‐partite meetings were valued for providing opportunities to share information and progress, and it is important to consider how positive relationships enhance these (Taylor‐Smith et al. 2023). Although the WBL portfolios were felt to be confusing when first introduced, an issue recognised by Minton and Lowe (2019), participants, similar to Konstantinou and Miller (2020) and Garnett (2020), recognised the important contribution they would make to AL development.

### 5.5 How, Where, and When—Structure and Organisation

Careful calibration of the academic, work‐based and placement strands of the training, including awareness of placement learning and overall progress, is required (Lester 2020). The means by which the AL becomes a competent professional in practice is thus more complex than in traditional undergraduate training, with the need for excellent time management skills (Baker 2019), including an ability to balance work and study demands (Bravenboer and Lester 2016), clearly identified. Mentors were central in providing models of workplace expectations of professionalism and facilitating theory‐practice application and learning opportunities within the workplace (Roberts et al. 2019) so are key. The expertise universities bring to designing and assessing higher‐level learning (Bravenboer 2016) was recognised, especially in relation to the WBL portfolio. Finally, face‐to‐face learning was supported as it alleviated isolation and had created a supportive peer group (Wenger 2015), with the collaboration and support of university tutors and workplace mentors adding to this (Nottingham and Yan 2023).

## 6 Clinical Implications and Future Research

### 6.1 Sustainability and Opportunity

The SLT apprenticeship has provided personal career progression opportunities that fit in with financial and home‐life factors for the AL participants researched in this study. This includes opportunities for accruing, applying and sharing learning in a continually dynamic way, meaning DAs may well hold advantages for adult‐learning, workforce development and clinical proficiency compared to traditional routes. This has important implications for the profession, though the question of retention and contribution to diversity/workforce development in the profession cannot be definitely answered at this early stage of delivery.

The apprenticeship would also appear to be a sustainable means of study for ALs, though they need to possess and/or develop excellent time‐management skills to simultaneously juggle university, placement and work‐based demands. Workload and additional placement requirements pose sustainability issues for mentors, though apprenticeship mentoring was felt to be both rewarding and to contribute to their own development.

DAs hold both opportunities and challenges for HEIs. Opportunities include synthesising academic teaching more closely to regular practice‐based learning and application. Challenges include implementing apprenticeship processes and devoting time to the support and development of strong AL‐mentor‐university partnerships.

### 6.2 Future Directions?

We are using the findings to develop our apprentice and mentor support and placement information, though this study contains information pertaining only to the first year of delivery of an SLT apprenticeship. We therefore hope to conduct a further study when this first cohort completes their 4‐year apprenticeship to capture stakeholder experience of the whole programme. We will therefore continue to interrogate the opportunities SLT apprenticeships provide to accrue, apply and share learning in a more dynamic way, recognising and harnessing the advantages for adult‐learning, workforce development and clinical proficiency apprenticeships may have over traditional routes, since the need for further research is indicated by this pilot study. Such future research should include a larger number of ALs, mentors and employers from different HEIs (several of whom have since launched their own SLT DA programmes) to increase the likelihood that the views and perspectives collected reflect overall SLT DA expectations and experiences.

### 6.3 Limitations

First of all, scale, timings, and staff vacancies in the research investigator team meant participant groups were restricted to the stakeholders most immediately involved with the SLT apprenticeship (ALs and mentors only), and student RA workload meant windows for data collection and analysis were both clearly defined and tight. Secondly, data were collected at the end of the first year of study, so it captures perspective and experience at an early stage of delivery, from this small cohort only (total possible participants = 38, actual = 18 for questionnaires, 9 for interviews). Conducting a follow‐up study at the end of the 4‐year programme, or a future larger‐scale study, may reveal different benefits and challenges to the current study's findings. Thirdly, all participants were either SLTs or AL SLTs in England; therefore, transferability of findings to other AHPs and geographical areas, where professional and apprentice standards and processes may be different, is not absolute. It is therefore recognised that issues such as sustainability and challenges for stakeholders cannot be identified unequivocally in this study.

### 6.4 Conclusion

The knowledge gained is being used to inform and enhance the DA offering and apprentice learning experience at the HEI where the study was conducted and is also relevant for other HEIs developing SLT apprenticeships and for stakeholders supporting practice relating to existing and future SLT and AHP apprenticeships. This is because the DA appears to provide an economically sustainable and practical progression opportunity for learners, including SLTAs, and because it provides SLT training that appears to be providing more opportunities for integrating theory and practice than traditional routes, though research further through the programme is required to fully ascertain this. Collaboration between the key stakeholders (AL, workplace and mentor, and HEI) is central to this, though challenges in terms of time and resources exist, including delivering DA and traditional programmes (HEIS) and placements (mentors) in parallel. Developing and sustaining SLT DAs therefore has implications for future workforce development and the profession.

## Disclosure

As authors, we disclose our interest in promoting degree apprenticeships (DAs) within the field of SLT, driven by a commitment to social justice and diversity within the profession, as a pragmatic response to workforce planning, and by our belief in the opportunity's DAs provide to accrue, apply and share learning in a more dynamic way. We acknowledge the potential influence of our perspectives on the study's findings and conclusions but also that we have endeavoured to maintain rigour, reflexivity and transparency throughout the research process and writing of this paper to ensure the integrity of our work.

## Conflicts of Interest

The authors declare no conflicts of interest. The authors alone are responsible for the content and writing of this article.

## Data Availability

The data that support the findings of this study are available from the corresponding author upon reasonable request.
